# Association between *Thiopurine S-Methyltransferase* Polymorphisms and Azathioprine-Induced Adverse Drug Reactions in Patients with Autoimmune Diseases: A Meta-Analysis

**DOI:** 10.1371/journal.pone.0144234

**Published:** 2015-12-03

**Authors:** Yue-Ping Liu, Han-Qing Xu, Ming Li, Xiang Yang, Shu Yu, Wei-Ling Fu, Qing Huang

**Affiliations:** 1 Department of Laboratory Medicine, Southwest Hospital, Third Military Medical University, Chongqing, China; 2 Department of Laboratory Medicine, 477th Hospital of PLA, Xiangyang City, Hubei Province, China; University of Utah, UNITED STATES

## Abstract

**Purpose:**

Azathioprine (AZA) is widely used as an immunosuppressive drug in autoimmune diseases, but its use is limited by significant adverse drug reactions (ADRs). Thiopurine S-methyltransferase (TPMT) is an important enzyme involved in AZA metabolism. Several clinical guidelines recommend determining *TPMT* genotype or phenotype before initiating AZA therapy. Although several studies have investigated the association between *TPMT* polymorphisms and AZA-induced ADRs, the results are inconsistent. The purpose of this study is to evaluate whether there is an association between *TPMT* polymorphisms and AZA-induced ADRs using meta-analysis.

**Methods:**

We explored PubMed, Web of Science and Embase for articles on *TPMT* polymorphisms and AZA-induced ADRs. Studies that compared *TPMT* polymorphisms with-ADRs and without-ADRs in patients with autoimmune diseases were included. Relevant outcome data from all the included articles were extracted and the pooled odds ratios (ORs) with corresponding 95% confidence intervals (CIs) were calculated using Revman 5.3 software.

**Results:**

Eleven published studies, with a total of 651 patients with autoimmune diseases, investigated associations between *TPMT* polymorphisms and AZA-induced ADRs, were included in this meta-analysis. Our meta-analysis demonstrated that *TPMT* polymorphisms were significantly associated with AZA-induced overall ADRs, bone marrow toxicity and gastric intolerance; pooled ORs were 3.12 (1.48–6.56), 3.76 (1.97–7.17) and 6.43 (2.04–20.25), respectively. *TPMT* polymorphisms were not associated with the development of hepatotoxicity; the corresponding pooled OR was 2.86 (95%CI: 0.32–25.86). However, the association in GI subset could be driven by one single study. After this study was excluded, the OR was 2.11 (95%CI: 0.36–12.42); namely, the association became negative.

**Conclusions:**

Our meta-analysis demonstrated an association of *TPMT* polymorphisms with overall AZA-induced ADRs, bone marrow toxicity and gastric intolerance, but not with hepatotoxicity. The presence of the normal *TPMT* genotypes cannot preclude the development of ADRs during AZA treatment, *TPMT* genotyping prior to commencing AZA therapy cannot replace, may augment, the current practice of regular monitoring of the white blood cell. Because of small sample sizes, large and extensive exploration was required to validate our findings.

## Introduction

Autoimmune diseases are a group of heterogeneous maladies in which the patient’s immune homeostasis becomes so deregulated that it mounts a destructive attack against the host’s tissues[1].Such diseases are characterized by the activation of T cells or B cells, or both, in the absence of an ongoing infection or other discernible cause[2, 3].The treatment strategies of such diseases include immunosuppressant—medication that alters thresholds of immune activation[3]. Azathioprine (AZA), a synthetic purine analogue, is widely used as immunosuppressive drug in autoimmune diseases, including rheumatoid arthritis (RA), autoimmune hepatitis (AIH), systemic lupus erythematosus (SLE) and autoimmune bullous diseases. Despite of its efficacy, AZA was documented for adverse drug reactions (ADRs), such as bone marrow toxicity (BMT), gastric intolerance (GI), pancreatitis, hepatotoxicity, etc.

The variable response to, and efficacy of, AZA are related to its pharmacogenetics. AZA is an inactive compound that must be metabolized to 6-thioguanine nucleotides (6-TGNs) to exert both the cytotoxic and therapeutic effects[4]. AZA is a pro-drug that is absorbed into the plasma and rapidly converted into 6-mercaptopurine (6-MP) via a glutathione-dependent process. Thiopurine S-methyltransferase (TPMT) is an important cytoplasmic enzyme catalyzing the methylation of 6-MP, competing with xanthine oxidase (XO) and hypoxanthine guanine phosphoribosyl transferase (HPRT) to determine the amount of 6-MP metabolized to 6-TGNs[5].The gene encoding for TPMT is subject to genetic polymorphisms that have been studied extensively. To date, a total of 37 mutations have been identified [6]. Approximately 4%-11% of individuals are heterozygous for a mutant *TPMT* allele and have intermediate TPMT activity; whereas approximately 1 in 300 individuals are homozygous or compound heterozygous and have very low or absent TPMT activity[7–9]. Individuals with intermediate TPMT activity accumulate 50% more 6-TGNs when compared with normal or high TPMT activity and thus at increased risk of AZA-induced ADRs [10]. Patients with deficient TPMT activity rapidly accumulate high doses of 6-TGNs, resulting in fatal bone marrow toxicity. Several clinical guidelines recommend determining *TPMT* genotypes or phenotypes before commencing AZA therapy [11–13].Drug label modifications for AZA approved by the U.S. Food and Drug Administration (FDA) also recommend pretesting, but does not mandate it[14]. The evidence base for these recommendations is unclear, particularly the crucial, direct evidence that pre-therapy *TPMT* measuring decreases BMT-specific mortality [15]. In addition, it is still controversial whether there is an association between *TPMT* polymorphisms and AZA-induced ADRs.

AZA, the pro-drug of 6-mercaptopurine, is also widely prescribed to patients with inflammatory bowel disease (IBD). Previous meta-analyses on association between *TPMT* polymorphisms and thiopurine-induced ADRs in patients with IBD were available [16–18]. However, to the best of our knowledge, there were no similar meta-analyses in patients with auto-immune disease. In the present study, we performed a meta-analysis with the purpose of gaining more insight into a possible association between *TPMT* polymorphisms and the common AZA-induced ADRs by evaluation of the literature on this subject. The finding of a significant association may become indirect evidence for pretesting *TPMT* genotype before commencing AZA therapy in patients with autoimmune diseases.

## Results

### Literature search outcome

**Fig 1:**
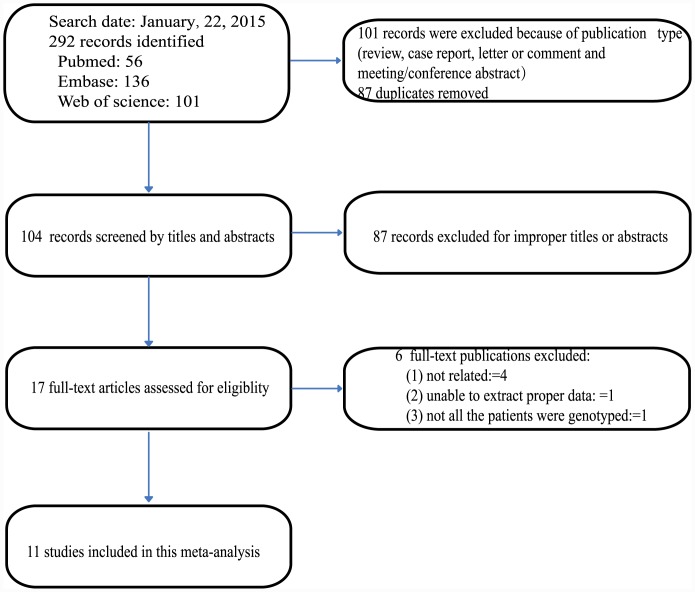
Flowchart describing the systematic literature search and study selection process.

With the aforementioned search strategy, a total of 292 potentially relevant records were retrieved. 101 records were excluded because of publication type (review, case report, letter or comment and meeting/conference abstract). 87 records were excluded because they were duplicates and another 87 records were excluded after reviewing the titles and the abstracts for improper titles or abstracts; 17 full-text papers were deemed to be relevant and were examined in detail. 6 full-text papers were excluded for the reasons described in Fig 1. Finally, 11 studies [19–29] met the inclusion criteria, and were included in this meta-analysis.

### Characteristics of included studies

A total of 11 studies with 651 patients with autoimmune diseases were included in our meta-analysis and the average number of patients per study was 59, ranging from 9[26] to 126[29]. A summary of the included studies is listed in Table 1.The earliest studies were reported in 1999 [19, 20], while the latest was in 2014 [29].4 studies[20, 23, 24, 29] were about the association between *TPMT* polymorphism and AZA-induced ADRs in SLE patients, 3 studies [21, 25, 26] focused on this association in AIH patients, another 3 studies [19, 22, 28] were talking about the association in patients with rheumatic diseases or RA while 1 study [27] concentrated this association in patients with autoimmune bullous diseases. Only one of the eleven included studies reported the occurrence of AZA-induced pancreatitis [25], but data were insufficient to calculate OR and corresponding 95%CI. 6[20–22, 25, 27, 28]of the 11 studies were from research in Caucasian populations of European ancestry, while the other 5[19, 23, 24, 26, 29]studies were from Asian populations. We can see that TPMT*3A is the most common mutant allele in Caucasians while TPMT*3C is the most common in Asians. TPMT*2 was a relatively rare variant allele, which was only found in one study[27]. As can be observed in Table 1, 8 studies determined TPMT*2,*3A, *3B and *3C alleles[19, 20, 22, 24, 26–29], 2 studies determined TPMT*3A, *3B and *3C[21, 25], while 1 study determined TPMT*2,*3A, *3B, *3C and *6 [23]. When all the studies were considered, including a total of 651 patients, three homozygous mutant genotypes were detected, with a frequency of approximately 1/217.

**Table 1:** Characteristics of 11 studies included in this meta-analysis*.

Author	Year	Country	Study Design	No. of Patients Included	Disease	*TPMT* Genotypes Determined	Profile of mutant TPMT polymorphisms	Dose of AZA	No. of Overall ADRs	NO. of BMT	NO. of Hepatotoxicity	NO. of GI	Ref.
Naughton, M A	1999	UK	CS	78	SLE	TPMT*2,*3A,*3B,*3C	1 Homo.*3A; 2 Het. *3A;1 Het.*3C	25–250 mg/day	17	13	3	1	[16]
Ishioka, S	1999	Japan	CS	36	Rheumatic Diseases	TPMT*2,*3A,*3B,*3C	3 Het. *3C	50 mg/day	NA	7	NA	NA	[17]
Langley, P G	2002	UK	CS	53	AIH	TPMT*3A,*3B,*3C	7 Het.*3A; 3 Het. *3B	50–100 mg/day	NA	3	NA	NA	[18]
Corominas, H	2003	Spain	CS	40	RA	TPMT*2,*3A,*3B,*3C	4 Het.*3A;1 Het.*3B	50–100 mg/day	6	2	NA	3	[19]
Jun, J B^#^	2005	Korea	CS	94	SLE	TPMT*2,*3A,*3B,*3C,*6	5 Het.*3C; 2 Het. *6	25–100 mg/day	23	17	1	4	[20]
Okada, Y	2005	Japan	CS	18	SLE	TPMT*2,*3A,*3B,*3C	2 Het.*3C	50 mg/day	NA	3	NA	NA	[21]
Heneghan, M A	2006	UK	CS	86	AIH	TPMT*3A,*3B,*3C	NA	100 mg/day	22	13	NA	NA	[22]
Tamori, A	2007	Japan	CS	9	AIH	TPMT*2,*3A,*3B,*3C	1 Homo.*3C	50 mg/day	NA	2	NA	NA	[23]
Bezier, M^#^	2008	France	CS	33	Autoimmune Bullous Diseases	TPMT*2,*3A,*3B,*3C	1 Het. *3C; 1 Het.*2	2.7mg/kg/day	NA	12	NA	NA	[24]
Tani, C	2009	Italy	CS	78	Rheumatic Diseases	TPMT*2,*3A,*3B,*3C	1 Homo.*3A; 1 Het.*3A	1.4mg/kg/day	NA	1	NA	NA	[25]
Chen, D	2014	China	CS	126	SLE	TPMT*2,*3A,*3B,*3C	4 Het. *3C.	1.4–2.0 mg/kg/day	44	34	4	4	[26]

*: The meta-analysis was performed on the studies looking at TPMT*3 family (including TPMT*3A, *TPMT* *3B and *TPMT* *3C).

^#^: All data being combined were the results from the same association model, thus Het*6 and Het*2 were not included in this meta-analysis.

CS: cross sectional; SLE: systemic lupus erythematosus; AIH: autoimmune hepatitis; RA: rheumatoid arthritis; AZA: azathioprine; TPMT: thiopurine S-methyltransferase; Homo.: homozygous; Het.: heterozygous; ADR: adverse drug reaction; NA: not available; GI: gastric intolerance.

In order to make the study clearer to readers, the definitions of AZA-induced ADRs are summarized as below: AZA-induced BMT markedly varied between studies, but the threshold for the number of leucopenia was generally set at 3–4×10^9^/L, and the number of neutrophils at 1.5×10^9^/L. The definitions of AZA-induced hepatotoxicity also differed between studies, with the level of alanine transaminase (ALT) set at >2 times the upper limit of normal (ULN). Gastric intolerance was defined as occurrence of any or a combination of the following: nausea, vomiting, dyspepsia and abdominal pain with normal amylase and normal abdominal ultrasound. Positive *TPMT* polymorphisms were defined as: with one or more mutant *TPMT* alleles (*TPMT* *3A, *TPMT* *3B and *TPMT* *3C).

### Meta-analysis outcomes

#### *TPMT* polymorphisms and AZA-induced overall ADRs

**Fig 2:**
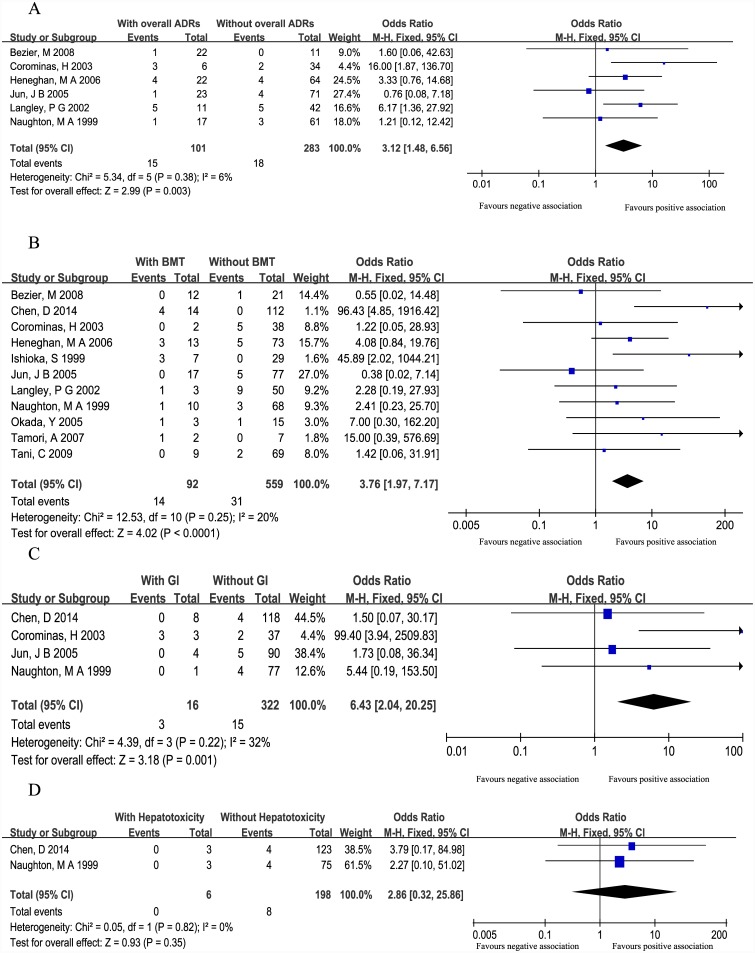
Forest plots of association between TPMT polymorphisms and AZA-induced overall ADRs (A), bone marrow toxicity (B), hepatotoxicity (C) and gastric intolerance (D). Total: total number of patients with or without ADRs. Events: number of patients with one or more mutant *TPMT* alleles (TPMT*3A, TPMT*3B and TPMT*3C) within the ADRs or no ADRs group.

6 studies [19, 21–23,25, 27], including 384 patients, analyzed the association between *TPMT* polymorphisms and overall ADRs. Of the 101 patients with overall ADRs, 15 (14.9%) patients were *TPMT* polymorphism positive and18 (6.4%) out of 283 patients without overall ADRs were *TPMT* polymorphisms positive. The pooled OR (3.12, 95%CI: 1.48–6.56) indicated a significant association between *TPMT* polymorphisms and AZA-induced overall ADRs (Fig 2A).

#### *TPMT* polymorphisms and AZA-induced BMT

All the included studies, with 651 patients, reported the association between *TPMT* polymorphisms and BMT. Of 92 patients with BMT, 14(15.2%) were *TPMT* polymorphisms positive, compared with 31(5.6%) of the 559 patients without BMT. There was a significant association between *TPMT* polymorphisms and BMT (pooled OR = 3.76, 95%CI = 1.97–7.17) (Fig 2B).

#### *TPMT* polymorphisms and AZA-induced GI

4 studies [20, 22, 23, 29] which included 338 patients, reported the correlation between *TPMT* polymorphisms and gastric intolerance. Of the 16 patients with GI, 3 (18.8%) patients were *TPMT* polymorphisms positive, compared with 15(4.7%) of the 322 patients without GI. The pooled OR (95%CI) was 6.43 (2.04–20.25) indicated a significant association between *TPMT* polymorphisms and AZA-induced GI (Fig 2C).

#### *TPMT* polymorphisms and AZA-induced hepatotoxicity

2 studies [20, 29], which included 204 patients, reported the correlation between *TPMT* polymorphisms and hepatotoxicity. Of the 6 patients with hepatotoxicity, no patients were *TPMT* polymorphisms positive, compared with 8 (4.0%) of the 198 patients without hepatotoxicity. The overall OR (2.86, 95%CI: 0.32–25.86) demonstrated that *TPMT* polymorphisms did not predict AZA-induced hepatotoxicity (Fig 2D).

### Subgroup analysis

#### Subgroup analysis according to ethnicity

**Fig 3:**
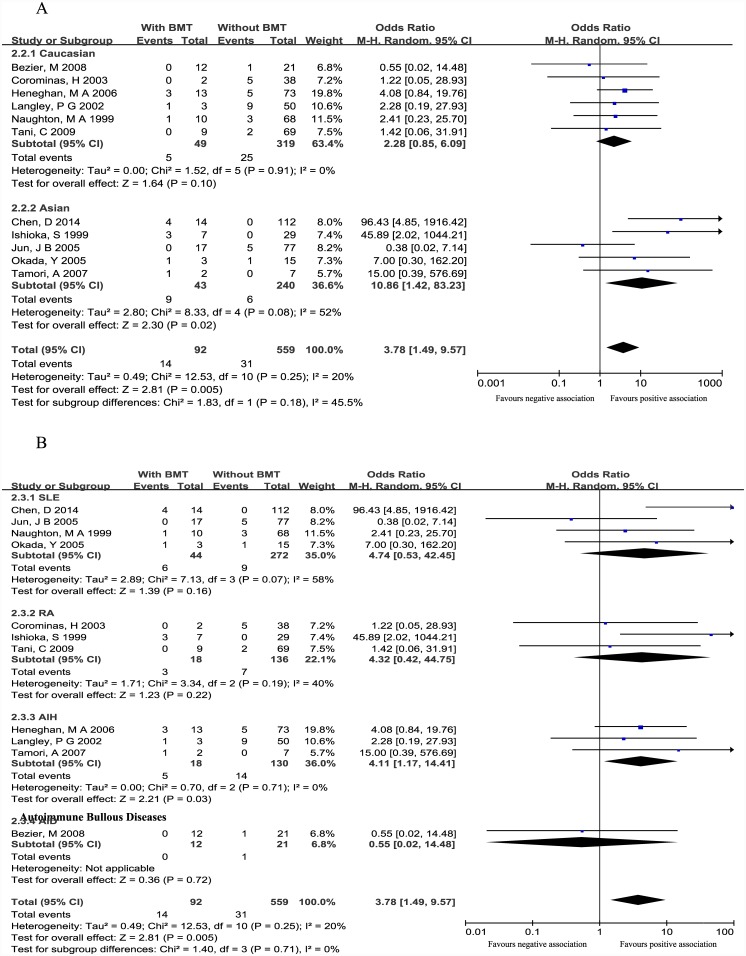
Forest plots of subgroup analysis according to ethnicity (A) and disease (B) in BMT subset. SLE: systematic lupus erythematosus. AIH: autoimmune hepatitis. RA: rheumatoid arthritis. Total: total number of patients with or without ADRs. Events: number of patients with one or more mutant *TPMT* alleles (TPMT*3A, TPMT*3B and TPMT*3C) within the ADRs or no ADRs group.

We performed subgroup analysis according to ethnicity in order to investigate whether the association signal differs among different ethnic origin. From the results, we can see that the pooled ORs (95%CI) of Caucasian population subgroup and Asian population subgroup in BMT subset were 2.28(0.85–6.09) and 3.78 (1.49–9.57), respectively. These results still showed a significant association between *TPMT* polymorphisms and AZA-induce BMT in Asian populations while the association in Caucasian populations was not significant (Fig 3A).

Because of small sample size, heterozygous and homozygous patients were grouped together as *TPMT* polymorphism positive. In order to better investigate the association between *TPMT* heterosigosity and AZA-induced BMT, an extra meta-analysis by excluding individuals with homozygosity genotypes of the genotyped *TPMT* polymorphisms was performed. The pooled OR (95%CI) of BMT subset was 3.47(1.78–6.79). This result was consistent with the original result, which indicated that *TPMT* heterosigosity was also associated with AZA-induced overall BMT.

#### Subgroup analysis according to disease

We also performed subgroup analysis according to disease in order to investigate whether the association signal differs among different disease sources. From the results, we can see that the pooled ORs (95%CI) of SLE subgroup, AIH subgroup, RA subgroup and autoimmune bullous diseases subgroup in BMT subset were 4.16 (1.59–6.88), 5.18 (1.36–19.69), 4.21 (1.25–14.15) and 0.31 (0.01–7.05), respectively (Fig 3B).

### Sensitivity analysis and publication bias

Sensitivity analysis was performed to examine the influence set by the individual study on the overall ORs by deleting each study once in every subset. Results in overall ADRs, BMT and hepatotoxicity subsets were consistent with the original results. However, the association in GI subset could be driven by one single study [22]. After this study was excluded, the OR (95%CI) was 2.31 (0.36–12.42), namely, the association became negative.

**Fig 4:**
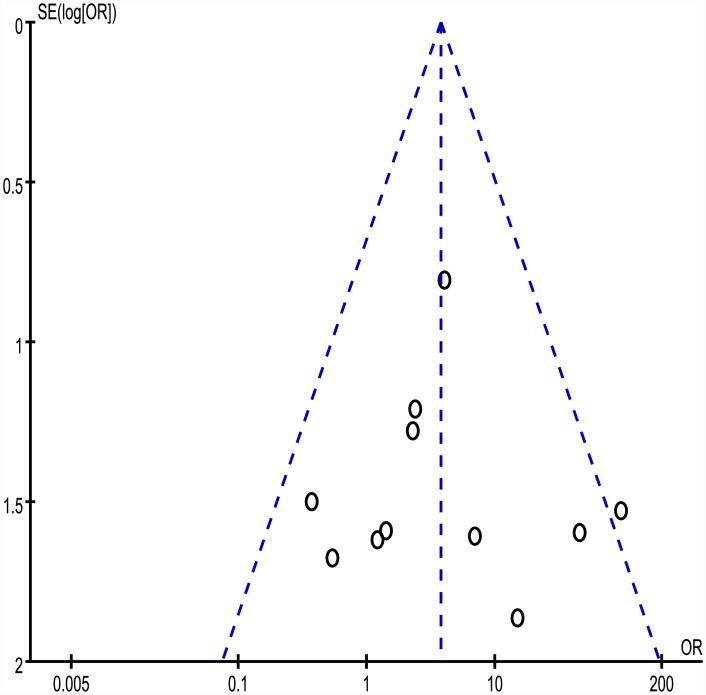
Funnel plot of BMT subset. The dotted vertical line indicates the overall OR. S.E. = standard error, OR = odds ratio. Each circle represents an eligible study.

As a recommendation, tests for funnel plot asymmetry should not be used when there are fewer than 10 studies in the meta-analysis [30], thus only funnel plot of BMT subset is shown in Fig 4. Egger’s test was used to provide statistical evidence of potential publication bias. The results did not suggest any evidence of publication bias.

## Discussion

AZA is widely used as immunosuppressive drug in autoimmune diseases. For instance, it has most often been prescribed as an alternative to cyclophosphamide and methotrexate (MTX) in SLE and RA [23], respectively. However, safety concerns do exist, because moderate to serious adverse events may occur. Gastric intolerance, bone marrow toxicity, hepatotoxicity and pancreatitis were among the most frequently reported clinically relevant adverse events. These events may be divided into dose-independent idiosyncratic reactions and dose-related, pharmacologically explainable toxicity [31].The results of our meta-analysis demonstrated that patients who were *TPMT* polymorphism positive were more likely to experience overall ADRs, BMT and GI, but not hepatotoxicity. However, several aspects have to be taken into consideration when interpreting the results of our study.

First, we identified that there have been a number of variants tested in the included studies for our meta-analysis. All the included studies determined the TPMT*3 family, while some studies tested additional variants, such as, a study of Korean population determined TPMT*6[23].TPMT*3A is the most common variant allele in Caucasian populations, while TPMT*3C is the most common mutant allele in Asian and African populations [32, 33]. TPMT*6 may be a potentially unique mutant allele within the Korean population, which was previously found in Korean children [34].Genotyping for the TPMT*3 family of variant alleles (TPMT*3A, TPMT*3B and TPMT*3C) will detect over 92% of low activity alleles and inclusion of TPMT*2 pushes this to over 95%[14]. However, all the studies tested the most common polymorphisms and these would miss rare variants, which may lead to the underestimation of the effect of *TPMT* polymorphisms on AZA-induced ADRs.

Second, *TPMT* polymorphism can explain a variable proportion of AZA-related ADRs, but in no way explain all episodes of ADRs [15]. In other words, the majority cases of ADRs were not *TPMT*-related, thus *TPMT* genotyping prior to commencing AZA therapy cannot replace, may augment, the current practice of regular monitoring of the white blood cell. Our study showed that *TPMT* polymorphism positive had specificity of 94.10% (526/559), but at the expense of a sensitivity of 16.30% (15/92) for predicting AZA-induced BMT. The specificity and sensitivity of *TPMT* polymorphism positive for predicting overall ADRs were 92.93% (263/283) and 14.85% (15/101).Several reasons may account for the gap between *TPMT* polymorphism and ADRs. First, up to 37 mutations in *TPMT* have been reported[6]. In included studies, 3 to 5 inactive *TPMT* alleles were investigated. We assume that patients with ADRs had one of the rare inactive *TPMT* alleles that were not examined. Another factor is the influence of variations in other genes, such as *hypoxanthine guanine phosphoribosyltransferase(HPRT)*[26]and *inosine triphosphate pyrophosphatase(ITPA)*[35].The development of AZA-induced ADRs is a multi-factorial event, caused by a co-influence of factors, other than variants in *TPMT*[36, 37], and a combined evaluation of the potential factors may enhance the correlation with ADRs. If possible, a genome-wide association study (GWAS) was required to investigate the associations between potential genes and ADRs. For instance, a study by Zalbala [37] reported variants associated with thiopurine-related BMT that was identified by GWAS. They indentified that rs372996 in *interleukia 6 singnal transducer (IL6ST)* gene and re3749598 in *follistatin-like 5 (FSTL5)* gene as new bone marrow toxicity susceptibility candidate genes after thiopurine treatment in IBD patients. The ORs (95%CI) were 3.41 (1.71–6.78) and 3.67 (1.68–8.01), respectively. Another GWAS association study published in Nature Genetics revealed variants associated with thiopurine-induced pancreatitis in patients with IBD [38]. Strong evidence of association within the class II HLA region were reported, with the most significant association identified at rs2647087 [OR (95%CI) 2.59 (2.07–3.26), p value of 2× 10^−16^].

Third, sample sizes of included studies were relatively small, ranging from 9 to 126 patients, which will increase uncertainty. The results of sensitivity analysis in our study showed that the association could be driven by one single study in GI subset. After this study was excluded, the association in GI subset became negative. In this study, three patients with *TPMT* polymorphism positive were observed to have GI reactions. Extensive exploration was required to support whether this was a chance phenomenon or not. Besides, a point of weakness in our study is the wide heterogeneity of patient cohort in terms of diagnosis. Although the pooled OR (95%CI) in BMT subset was 3.76 (1.97–7.17), subgroup analysis in BMT subset showed the pooled ORs (95%CI) of SLE subgroup, AIH subgroup, RA subgroup and autoimmune bullous diseases subgroup in BMT subset were 4.74 (0.53–42.25), 4.32 (0.42–44.75), 4.11 (1.17–14.41) and 3.78 (1.49–9.57), respectively.

Fourth, disease-related BMT episodes are often difficult to distinguish from those caused by drugs, such as BMT is a feature of SLE disease activity. The study by Naughton et al. [20]detected that 13 patients with BMT, however subsequent investigations found that, in three of these cases, the BMT was disease related, while AZA was strongly implicated in the remaining 10 cases.

Fifth, there is no doubt in the literature that patients who are homozygous or compound heterozygous for a variant allele confer a very high-risk of early severe BMT. This was confirmed by our study; as 2 out of the three homozygotes detected in all the 651 patients experienced early, severe leucopenia requiring hospital management. Such susceptible patients can be identified by *TPMT* genotyping prior to commencing AZA therapy, thus avoiding potentially fatal consequences. Prospective studies are required, however, to explore the cost-effectiveness of this approach due to low frequency of homozygous individuals.

Sixth,because of the life threatening nature of AZA-induced BMT, pretesting for *TPMT* genotype before the initiation of AZA therapy has increasingly been accepted clinically. Several guidelines recommend determining *TPMT* status before AZA therapy. However, these recommendations are considered to be premature from an evidence-based perspective, due to the absence of direct and crucial evidence that *TPMT* pretreatment testing decreases BMT-specific mortality [15].

In conclusion, our meta-analysis demonstrated an association of *TPMT* polymorphisms with overall AZA-induced ADRs, bone marrow toxicity and gastric intolerance, but not with hepatotoxicity. Because of small sample sizes and wide heterogeneity of patient cohort in terms of diagnosis, large and extensive exploration was required to support whether these findings were chance phenomena or not. *TPMT* polymorphism can explain a variable proportion of AZA-related ADRs, but in no way explain all episodes of ADRs. In other words, the presence of the normal *TPMT* genotype cannot preclude the development of ADRs during AZA treatment, *TPMT* genotyping prior to commencing AZA therapy cannot replace, may augment, the current practice of regular monitoring of the white blood cell.

## Materials and Methods

### Literature search strategy

Medline (using PubMed as the search engine), The Excerpta Medica Database (Embase) and Web of science were searched to identify relevant publications published in English with an end date of January 22, 2015. Only human-related literature was searched. We employed both MeSH terms and free text words (in Title/Abstract fields) for ‘*TPMT*’ or ‘thiopurine S-methyltransferase’ or ‘thiopurine methytransferase’ **AND** ‘azathioprine ‘ or ‘imuran’ or ‘6-mercaptopurine’ **AND** (‘autoimmune diseases’ **OR** ‘SLE’ or ‘systemic lupus erythematosus’ or ‘lupus erythematosus disseminatus’ or ‘libman scks disease’ **OR** ‘RA’ or ‘Rheumatoid Arthritis’ **OR** ‘AIH’ or ‘Autoimmune Hepatitis’). We also performed a manual search of the references listed in the articles identified in the search for additional eligible studies. The search was conducted independently by two reviewers (YPL and HQX).

### Inclusion and exclusion criteria

The abstracts and full texts were read independently by the two reviewers (YPL and HQX). The following inclusion criteria were used: 1) studies that compared *TPMT* polymorphisms between with-ADRs and without-ADRs in patients with autoimmune diseases; 2) articles published in English and being human-related were included; 3) expert opinions supported by a preliminary literature review indicated that there was likely to be very few randomized, controlled trials (RCTs) on this topic; therefore, any study design (cross-sectional cohort, prospective cohort and case control studies) were included in this meta-analysis [39]; 4) all patients included in this meta-analysis were genotyped for *TPMT* polymorphisms; 5) studies that tested at least TPMT*3A, *TPMT* *3B, *TPMT* *3C, regardless of whether they tested additional mutant alleles. Studies on non-autoimmune diseases patients were excluded. Reviews, letters, comments, and conference abstracts were also excluded because of limited data. Further, publications identified as duplicates were excluded.

### Data extraction strategy

Two reviewers (YPL and HQX) independently extracted relevant data from each eligible study. The following data were collected: author’s name, publication year, country, study type, number of enrolled patients, disease, profile of mutant *TPMT* polymorphisms, AZA dose, number of patients that were mutant-type *TPMT* with and without an ADR, *TPMT* polymorphism type, number of homozygous mutant-type *TPMT*, and definitions of ADRs. Disagreements between reviewers were resolved by discussion or by consensus including a third author (QH).

### Statistical analysis

In order to make results of the present meta-analysis more robust and reasonable, all data being combined were the results from the same association model. In addition, TPMT*3B is usually in tight linkage disequilibrium with the *3C SNP, resulting in the common allele, *3A[40]. Thus, the meta-analysis was performed on the studies looking at TPMT*3 family (including TPMT*3A, *TPMT* *3B and *TPMT* *3C). OR and 95% CIs were calculated to mainly evaluate the strength of associations between TPMT*3A/TPMT*3B/ TPMT*3C and AZA-induced ADRs. Not all studies reported all ADRs analyzed in this meta-analysis, and so only studies that reported the adverse events of interest were analyzed for the association between *TPMT* polymorphisms and that adverse event. The included studies displayed heterogeneity concerning diagnosis, the time to onset of AZA-induced ADRs, definitions of the ADRs, and study designs. The degrees of included studies’ heterogeneity were explored using the chi-squared test of heterogeneity, and inconsistency index (I^2^). Considering the low statistical power of these tests, a p-value of <0.10 or an I^2^>50% was defined as significant heterogeneity. ORs from different groups were combined using fixed or random effects models, which depends on the absence or presence of significant heterogeneity.

Subgroup analysis was conducted according to ethnicity or disease in order to investigate whether the association signal differs among different ethnic origin or different diseases.

Sensitivity analysis was performed to assess the stability of the results; namely, a single study in the meta-analysis was deleted each time to reflect the influence of the individual data set to the overall OR. Publication bias was assessed by visual inspection of the funnel plot for symmetry, and formal statistical testing using the Egger test. The meta-analysis was conducted using RevMan 5.3 software.

## Supporting Information

S1 Checklist PRISMA 2009 Checklist. (DOC)

S2 Checklist Genetic checklist. (DOCX)
